# The role of social media in enhancing health awareness, social support and educational progress among university students in Oman during the COVID-19 pandemic: a cross-sectional survey

**DOI:** 10.3389/fsoc.2026.1873019

**Published:** 2026-09-15

**Authors:** Emad Farouk Saleh, Wafa Said Almamari, Saudi Mohamed Hassan Abdelreheem, Ahmed Thabet Helal Ibrahim, Magdy Mohammed Mustafa, Hosni Ibrahim Abdelghani

**Affiliations:** 1 Sociology and Social Work Department, Sultan Qaboos University, Seeb, Oman; 2 College of Arts and Social Sciences, Sultan Qaboos University, Muscat, Oman

**Keywords:** academic programs, COVID-19 pandemic, educational needs, health and social awareness, social media, social work practice, Sultanate of Oman

## Abstract

This study examined the role of social media in promoting health awareness, strengthening social support, and maintaining educational continuity among university students during the early stage of the COVID-19 lockdown in Oman. It also explored the major challenges associated with social media use during this unprecedented public health emergency. A descriptive-analytical design was employed using an online survey administered to 380 undergraduate students at Sultan Qaboos University during the suspension of face-to-face teaching in April and May 2020. The findings indicate that social media played an important role in enhancing students’ awareness of COVID-19-related health issues, with a high overall awareness score (M = 40.92, SD = 5.47; relative strength = 85%). Students also reported substantial social benefits (M = 50.15, SD = 8.19; relative strength = 84%), particularly in strengthening social responsibility, maintaining communication with family and friends, and reducing the effects of social isolation. In addition, social media contributed positively to educational continuity during the lockdown (M = 30.80, SD = 7.89), especially by facilitating access to learning materials, communication with instructors, and the submission of assignments. Despite these benefits, students identified several important challenges (M = 34.02, SD = 5.67; relative strength = 77%), including the spread of misinformation and rumors, unreliable online content, high internet costs, and technical and connectivity problems. The findings highlight the value of social media as a complementary tool for health communication, social connectedness, and educational continuity during public health emergencies. They also provide insights that may inform digital communication strategies in higher education and public health campaigns. Although the study did not specifically investigate social work students or practitioners, the findings may also inform the development of digital competencies and electronic social work education for crisis preparedness. Further research should examine the role of social workers and social work education in emergencies and disaster contexts.

## Introduction

Since the creation of social media platforms, arguably as early as 1997 or 2002/3, but indisputably with the launch of Facebook in 2005, they have gained widespread and worldwide usage, being used by approximately 60% of the world’s population, or around five billion users (United Nations, 2023). The advantages they offer are numerous. Social media is fast and efficient and provides an abundance of easily accessible and exchanging information. It also allows communication, participation in data- and information-sharing, the formation of remote work teams, access to services provided by multiple websites and participation in obtaining information technology; its platforms can also be utilized in education systems and processes (Drahošová and Balco, 2017).

The growth of social media, then, has provided a variety of ways for people to communicate, share news and make information more accessible (Merchant and Lurie, 2020; Laranjo et al., 2015). Although the information disseminated may not always be accurate. Social media can also offer positive socioeconomic and climate values (Gluskin et al., 2014).

The first confirmed COVID-19 cases in Oman were reported in February 2020. As infections increased rapidly, the government implemented a series of public health measures, including lockdowns and restrictions on face-to-face activities. In response, Sultan Qaboos University suspended on-campus teaching in April 2020 and shifted to remote learning. This unprecedented situation increased students’ reliance on social media and digital platforms for accessing health information, maintaining social connections, and supporting their educational activities. These circumstances provided the context for the present study, which examines the role of social media during the initial stage of the COVID-19 pandemic in Oman (Ministry of Health Oman, 2025).

Throughout the world, social media played a crucial role in disseminating timely information about COVID-19, combating misinformation, and promoting public health awareness. It also enabled individuals, organizations, and governments to communicate and coordinate responses during the pandemic (Mohsen et al., 2022; Abuhashesh et al., 2021). These functions can be understood through the Uses and Gratifications Theory, which suggests that individuals actively select media platforms to satisfy specific information, educational, and social needs, particularly during periods of uncertainty and crisis. Likewise, Social Support Theory explains how online communication platforms facilitate emotional, informational, and social support, helping individuals maintain social connectedness and cope with stressful situations. During the COVID-19 pandemic, social media therefore served not only as an information source but also as a means of sustaining interpersonal relationships and educational continuity. These platforms supported public health campaigns by promoting preventive behaviors, disseminating vaccine-related information, and enabling health authorities to monitor public concerns and community needs through social media analytics (Yigitcanlar et al., 2020). During the early stages of the COVID-19 pandemic, the unprecedented nature of the outbreak and the rapidly evolving scientific evidence made timely public communication challenging in many settings. Consequently, many individuals turned to social media to obtain real-time information, although the quality and reliability of such information varied considerably (La et al., 2020).

While there were many advantages to the use of social media during the pandemic, it also resulted in several challenges. One of the most significant was the spread of misinformation and of conspiracy theories about the virus; these related especially to vaccines and vaccinations. Some users exploited social media to spread rumors and conspiracy theories that undermined public health efforts; the platforms struggled to curb the spread of false information. People’s increased reliance on social media during the pandemic also created threats to privacy and cybersecurity, with hackers exploiting vulnerabilities in the platforms to steal personal information or spread malware. Despite these challenges, social media has continued to play a significant role in promoting public health, advancing educational progress and alleviating social isolation in the post-pandemic world; however, it will be essential to address the challenges it poses while maximizing its potential benefits. This will require ongoing efforts to combat misinformation, protect privacy and security, and promote responsible social media use.

In Oman and elsewhere, COVID-19 had a major impact on students at all levels, not only on their health but also on their educational and social experiences. At the beginning of the pandemic in particular, when universities transitioned, often abruptly, to remote learning, students faced increased isolation and uncertainty. Social networking platforms quickly emerged as a potential tool that could promote health awareness, boost social interaction and – especially – support educational efforts during the pandemic (Baltacı, 2019). With schools and universities closed or operating at reduced capacity, educators had to adapt to remote learning methods. Social media provided a platform where teachers and students could communicate and collaborate online, access learning materials, and share resources. It also enabled educators to maintain social connections with and support for their students during a time of physical distancing and isolation.

As the pandemic progressed, an increasing number of studies examined the impact of COVID-19 on university students, highlighting the important role of social media in facilitating health communication, supporting online learning, and maintaining social connectedness. However, findings across studies were not entirely consistent, as researchers also reported challenges related to misinformation, excessive media exposure, and unequal access to digital resources. Furthermore, despite the growing international evidence, limited research has examined these issues in the Omani context, particularly regarding how social media contributed simultaneously to health awareness, social support, and educational continuity among university students during the early stage of the COVID-19 pandemic. This gap provided the rationale for the present study.

The aim of this research study, then, was to identify the role played by social media in increasing the awareness and information of university students about COVID-19, and its role in supporting them socially and educationally, specifically at the start of the outbreak.

The main objective was broken down into several research questions, namely:

1. How can social media provide information and raise awareness among students about the COVID-19 virus in general, its symptoms, and ways to prevent its spread?
2. How can social media platforms help students maintain social connections during the pandemic, provide emotional support, and alleviate feelings of isolation during lockdowns and when other restrictive measures are imposed?
3. How were social media platforms used to support remote learning during the COVID-19 pandemic?
4. How did students perceive remote learning through social media platforms during the COVID-19 pandemic.
5. What challenges prevent students from fully benefiting when using social media platforms for health, social and educational purposes during the pandemic?

The study also aimed to use the results to propose suggestions for developing an action plan which would enable social workers, students and professionals to use these networks more effectively to confront the COVID-19 pandemic and its aftermath, as well as future crises and disasters.

## Materials and methods

A cross-sectional descriptive survey design was adopted to achieve the objectives of the study. A social survey was conducted among undergraduate students enrolled at Sultan Qaboos University (SQU) during the initial stage of the COVID-19 pandemic, when the university had suspended face-to-face teaching and shifted to remote learning. The study population comprised 16,018 undergraduate students enrolled at SQU during the 2019/2020 academic year. Data were collected between 22 April and 26 May 2020, corresponding to the first COVID-19 lockdown in the Sultanate of Oman. An electronic questionnaire was distributed through the official university email system to all undergraduate students.

Two reminder emails were sent during the data collection period to emphasize the importance of completing the questionnaire and to encourage voluntary participation.

A total of 380 questionnaires were received, representing an adequate sample size according to Steven Thompson’s sample size formula. Conducting the survey during this period enabled the study to capture students’ perceptions of the role of social media during the initial stage of the pandemic.

### Participants and procedure

#### Sample size calculation

The minimum required sample size was calculated using Thompson’s finite-population sample size formula (Thompson, 2012).


$$n=\frac{Nz^{2}p(1-p)}{d^{2}(N-1)+z^{2}p(1-p)}$$

Where N represents the population size, z represents the 95% confidence level, p represents the expected proportion in the population, and d represents the margin of error. Using N = 16,018, z = 1.96, *p* = 0.50, and *d* = 0.05, the minimum required sample size was approximately 376 students. 380 questionnaires were received, and four incomplete questionnaires were excluded, resulting in a final sample size of 376 students.

A total of 380 questionnaires were returned, yielding a response rate of 2.37%. After excluding four incomplete questionnaires, the final analytical sample consisted of 376 students, thereby meeting the minimum required sample size for the study the participants registered voluntarily, and the data were collected during the period between 22 April and 26 May2020. Demographic data from the survey shows that 42.6% of the participating students were male and 57.4% female; their mean age was 21.27 years. The students were from most of the governorates of the Sultanate, as shown in Table 1 below, with the highest percentage, 26.3%, coming from Muscat governorate, which is the capital of Oman and the most highly populated governorate in the country. Table shows the demographic distribution of the participating students, including the governorates where they lived.

**Table 1:** Student gender distribution according to Omani governorates.

Governorate	Gender		Total
	Male	Female	
Muscat	7.4%	18.9%	26.3%
Al Buraimi	0.0%	0.0%	0.0%
Al Wusta	0.0%	0.0%	0.0%
Al Dakhiliya	9.6%	9.6%	19.1%
Dhofar	1.1%	2.1%	3.2%
Musandam	1.1%	0.0%	1.1%
Al Batinah North	13.8%	6.4%	20.2%
Al Batinah South	2.7%	16.0%	18.6%
Al Sharqiya North	1.1%	2.4%	3.5%
Al Sharqiyah South	3.2%	0.0%	3.2%
Al Dhahira	2.7%	2.1%	4.8%
Total	42.6%	57.4%	100.0%

Although the data were collected during the first lockdown period of the COVID-19 pandemic (April–May 2020), the findings remain scientifically valuable because they capture students’ experiences and perceptions during a unique and unprecedented period in which social media played a critical role in information dissemination, awareness raising, and supporting remote learning. Examining these experiences provides important insights into the role of social media during public health crises and offers lessons that can inform future emergency preparedness and the use of digital platforms in education and communication.

#### Ethics

The authors ensured that all participants were fully informed about the study and that their participation was entirely voluntary. Informed consent was obtained from the students before they proceeded to complete the study instrument. No personal data that could potentially reveal participants’ identities to readers were requested.

#### Questionnaire

The questionnaire began by asking students to give their gender and age; to state the number of hours a day they used social media, and to identify the devices they used most frequently during the period of remote learning. The main part of the survey consisted of seven sections using a modified 4-point Likert scale for each; the sections contained between 7 and 15 points each, and investigated the following issues:

1. The social media students used during the COVID-19 university lockdown (and the degree of use of each).
2. The sources of information about COVID-19 they relied on (and their degree of reliance on those sources).
3. Their level of trust in each source of information listed (and their level of confidence).
4. The role of social media in raising their awareness of key areas of information about the pandemic (and their degree of approval of the information on each area).
5. The social benefits they obtained from their use of social media (and the degree of benefit they obtained).
6. How they used social media to carry out various academic/educational tasks (and how well each task could be satisfactorily carried out).
7. Challenges they encountered in using social media during the COVID-19 pandemic (and the extent of those challenges).

The questionnaire was developed specifically for this study and used four response categories that varied according to the axis: high, medium, low, non-use/non-reliance/lack of trust, or strongly agree, agree, somewhat agree, and disagree. The questionnaire was also shown to eight professors of sociology and social work to verify the content validity, and their comments were taken into account when preparing the final version.

The survey concluded with two open-ended questions:

1. How did social media be better used to reduce the risks associated with the pandemic?
2. Can you suggest an idea or voluntary social initiative that you hope will be implemented in your community or place of residence to improve the situation created by the pandemic?

#### Open-ended answer analysis

The responses to the two open-ended questions were analyzed using qualitative content analysis. The responses were read and coded according to their main ideas, and similar responses were then grouped into frequent categories. The most prominent categories were then summarized, and representative quotes were selected to illustrate the findings.

After the data were processed, the reliability statistics of the tool were measured using Cronbach’s Alpha coefficient. The value given was 0.91, which indicates excellent internal consistency. The independent samples t-test was used to measure differences by gender and place of residence, and one-way ANOVA was used to measure differences by household income, with the LSD test used to identify significant differences at the *p* < 0.05 level.

## Results

Field data analysis showed that, during the period of suspended classes, participating students spent a mean of 6.87 h daily using social media; this time period included academic work and also general communication, information-browsing, and entertainment.

### Data analysis

The results showed that students relied on various types of electronic devices to access social media applications and networks. Table 2 presents the frequency with which different devices were used during the suspension period.

**Table 2:** Most common electronic devices used by students during the period of university suspension.

Media and electronic devices		Frequency	Percent
Valid	Smartphone	338	89.9
	Laptop	24	6.4
	Smart TV	8	2.1
	Smartphone, laptop, smart TV	6	1.6
	Total	376	100.0

Smartphones were indisputably the most commonly used device, at 89.9%; an earlier study among students at the same university had also found that smartphones were far and away the most commonly used devices to access social media (Saleh and Al-Rawas, 2015). This was followed by laptops with a percentage of 6.4% and smart TVs with a percentage of 2.1%; and only 1.6% reported using more than one device to access social media. The predominance of smartphones is unsurprising, as most of the current generation of smart mobile phones are lightweight, easy to use, and constantly connected to the internet; their digitally native owners can thus use multiple social media applications and networks without interruption.

### Survey section 1: social media platforms used during the early stages of the spread of COVID-19

It is important to note the distinction between the first three sections of the survey questionnaire. This first section deals with what social media sources students used, in general, during the early stages of the pandemic, while the second investigates which sources they used specifically to gain information about COVID-19, and the third queries their levels of confidence about the COVID-related information offered by these sources.

Table 3 presents the social media platforms used during the early stages of the pandemic; that is, which social media they used in general.

**Table 3:** Social media platforms used during the emergence of (COVID-19).

Social media platforms	Mean	Std. deviation	Relative mean	Ranking
YouTube	2.91	0.94	73%	1
Instagram	2.83	1.10	71%	2
Twitter(X)	2.37	1.05	59%	3
Snapchat	2.33	1.08	58%	4
Facebook	1.17	0.43	29%	5
WhatsApp	0.74	2.11	18%	6

The results show that the most commonly used platforms were YouTube, which ranked first with a relative mean usage of 73.0%, followed by Instagram (71%), Twitter (X) (59%), and Snapchat (58%). Facebook ranked fifth with a relative mean usage of 29%, whereas WhatsApp was reported as the least frequently used platform, with a relative mean usage of 18%. This finding contrasts with previous studies conducted in Oman. For example, the Digital Indicators Report for the Information and Communications Technology sector in Oman identified WhatsApp as the most widely used social media application, followed by YouTube (Ministry of Technology and Communications, 2020). Similarly, Saleh et al. (2023) reported that WhatsApp was the most commonly used social networking application among Omani citizens, with a relative mean usage of 87%. The present study did not investigate the reasons for this discrepancy; therefore, no definitive explanation can be provided. Future research is needed to examine the factors that may account for this difference.

### Survey section 2: sources of information used for COVID-related information during the early spread of pandemic

It may now be hard to recall how alarming and unprecedented were the arrival and early spread of the COVID-19 pandemic. There was little information available to respond to people’s questions and concerns, and even information from official sources changed very rapidly as the pandemic escalated and fatalities increased.

The World Health Organization (WHO) and the Centers for Disease Control and Prevention (CDC) were and remain the main sources of accurate and reliable information about the pandemic and of recommendations for prevention and treatment, but at the time they were frequently forced to adapt information and advice as amounts of data increased. Their reports and notifications were followed by reputable media and by ministries of health and other government entities, as well as by other international health organizations like the Red Cross/Crescent and the Bill and Melinda Gates Foundation.

Given the suddenness and severity of the pandemic, it was vital that people ignore rumors and rely on reliable sources providing accurate information (BBC, 2020). At the same time, however, throughout the pandemic but especially at the beginning,

There was a great deal of inaccurate and misleading information circulating on social media, some of it at least attempting to cheat people and/or gain access to their private and financial information.

Figure 1 presents the hierarchy of information sources actually used by the research participants in the initial stages of the pandemic. As shown in Figure 1, participants used a variety source to obtain information about the pandemic. It should be noted that the frequency of using these sources does not necessarily reflect participants’ levels of trust them. It must also be noted that participants’ levels of use may not automatically reflect levels of their trust and confidence. Social media in general was the major source of information about the pandemic, at 80%, followed by general internet browsing, which had a relative mean of 73%. Specific government websites also played a significant role in providing information, at 71%, followed by Omani government TV channels at 67%. Global and local news channels were used at 59 and 58%, respectively. More private communication sources like email and SMS were identified as the least used sources of information, with relative means of 54 and 48%.

**Figure 1:**
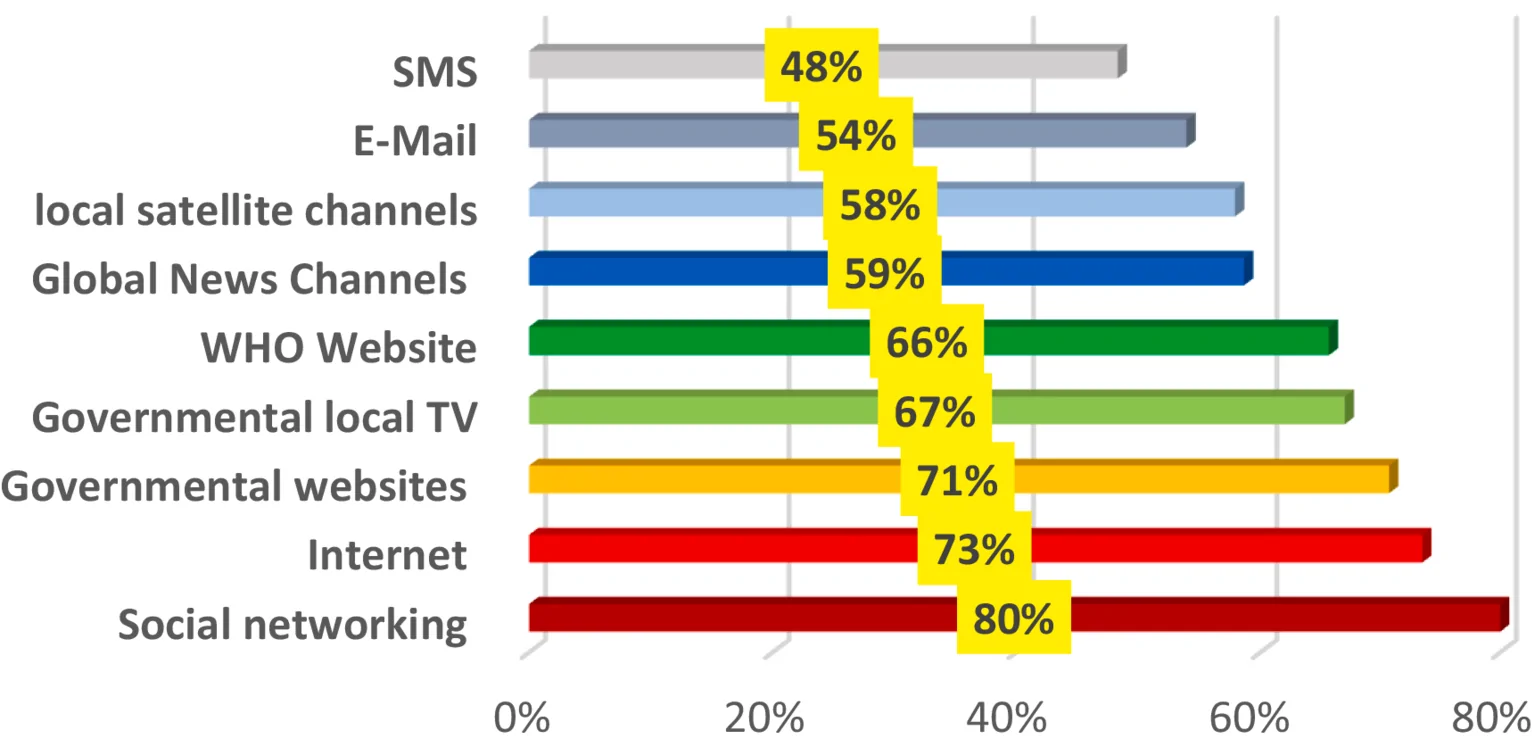
Sources of information about COVID-19 during the initial stages of pandemic.

These findings are very similar to those of a later investigation by Saleh et al. (2023), which found that, in the initial phases of the pandemic, social media played a crucial part in disseminating information, though it must be noted that this was true for both reliable and unreliable information. It cannot, however, be assumed that social media would continue to play this role to the same degree in the later stages of the pandemic.

### Survey section 3: students’ trust in sources of information about the COVID-19 pandemic

A previous study found that trust in health information sources was associated with increased adherence to recommended preventive behaviors during the COVID-19 pandemic (Figueiras et al., 2021).

As seen in this Table 4, at the beginning of the pandemic and the imposition of distance learning, student participants obtained information about COVID-19 from various sources. However, the levels of trust in these sources were by no means equal, as shown in Table 5 below.

**Table 4:** Degrees of confidence in sources of information about Covid-19.

Information sources	Mean	Std. deviation	Relative mean	Confidence level	Ranking
Omani government TV	3.66	0.59	92%	Very strong	1
Omani government websites	3.59	0.64	90%		2
WHO website	3.59	0.75	90%		3
Global news channels	3.29	0.73	82%		4
Local satellite channels	3.23	0.73	81%		5
SMS	2.78	0.92	69%	Strong	6
Social media networks	2.76	0.79	69%		7
General websites	2.69	0.77	67%		8

**Table 5:** The role of social media in raising health-related issues about Covid-19.

Pandemic-related issues	Mean	Std. deviation	Relative mean	Awareness level	Ranking
Awareness of prevention precautions to be followed, such as hand washing and social distancing	3.68	0.61	92%	Very strong	1
Raising awareness of how the pandemic spreads	3.66	0.57	92%		2
Recognize ways to prevent the spread of the pandemic	3.62	0.55	91%		3
Raising awareness about the pandemic in general	3.62	0.64	91%		4
Raising awareness of the danger of the virus at a personal level	3.61	0.61	90%		5
Monitoring the spread of the virus at the local level	3.57	0.74	89%		6
Awareness of the measures taken by the government to fight the pandemic	3.54	0.66	88%		7
Knowledge of symptoms of being infected with the virus	3.54	0.61	88%		8
Knowing where to access healthcare services in case of emergency.	3.36	0.81	84%		9
Correcting some of my misconceptions about the virus.	3.34	0.75	84%		10
Following the global spread of the virus.	3.29	0.86	82%		11
Being misled by inaccurate information about the virus.	2.09	0.89	52%	Average	12

The “strong/very strong” levels of confidence shown in the table followed the criteria used by Saleh and Al-Rawas (2015) to describe the levels of relative strength of each finding: less than 50% was labeled weak, 50% to 64% was average, 65% to 79% was seen as strong, and a relative mean over of 80% or more was labeled very strong. Overall, then, and interestingly, participants had greatest trust in sources from the Omani government, with government television channels ranked first with a trust level of 92%, and government websites at 90%, identical to the trust in the World Health Organization website. Trust in global and local news channels was slightly lower, but still very strong. The lowest levels of trust were in information from SMS, social media and general websites, though these were still strongly trusted at 69%, 69%, and 67%, respectively. These findings were consistent with those in the study by Saleh et al. (2023), which showed that the participants trusted Omani government sources more than any others. An interesting point here is that although survey section 2 showed that social media networks were the most used sources of information on COVID-19, they were among the least trusted, a fact whose implications will be explored in the Discussion section.

### Survey section 4: the role of social media in raising health awareness about the COVID-19 pandemic

Social media have often been used to improve health awareness, and this section explored how important a role they played in sharing vital information and educating the student participants about Covid-19 in its early stages. This survey section consisted of 12 statements aimed at measuring their perception of this; the results can be seen in Table 5. The first eleven of the twelve statements concern the positive input made by social media; only the last has a different focus and refers to its negative impact.

The results presented in Table 5 showed that social media made a very significant contribution to raising awareness of all aspects of the pandemic: how the disease is transmitted, infection-prevention precautions, the symptoms of the virus, the extent of its local and global spread, government measures being taken to deal with it and how to access health services if needed. The mean average degree of responses to all the statements except the last was a relative strength of 85%, indicating that this role was very strong (80% or more means very strong).

Overall, then, despite the survey being conducted at the early stage of COVID-19, results showed that social media networks greatly helped students understand all aspects of the pandemic (M = 40.92, SD = 5.47, relative strength = 85%) and served to correct their misconceptions about it. Results for the last statement, however, revealed that social media networks did also play a negative role and spread confusion and false information. This is understandable, as social media is accessible to all members of society, and many exchange written, audio, and visual messages without verifying the accuracy of the content they are sharing. The results for this statement reached 2.09 out of 4 degrees and thus had a relative mean of 52%; this indicates a medium level according to the criteria used in this research and was much lower than the results for all the positive statements, but still significant.

### Survey section 5: social benefits of social Media at the Beginning of the spread of COVID-19 in Oman

Since their creation, social media has become an essential and worldwide platform for social interaction and communication among individuals and communities. In the exceptional circumstances created by the outbreak of the pandemic, these platforms became a crucial way not only to obtain accurate and reliable information about the virus but also to maintain communication with others. At a time of social isolation and widespread quarantining, these networks provided many social benefits for individuals and communities, promoting communication, providing psychological support, and allowing people to communicate with healthcare professionals to obtain information, as well as providing entertainment activities to alleviate the psychological pressures of isolation. The students’ responses to the questions about the perceived social benefits of social media are shown in Table 6 below.

**Table 6:** Social benefits of social media at the beginning of the spread of COVID-19.

Social benefits	Mean	Std. deviation	Relative mean	Social benefits level	Ranking
Helping me to develop a sense of social responsibility towards family and community.	3.67	0.55	92%	Very strong	1
Informing me about precautions to avoid spreading or contracting COVID when dealing with others.	3.64	0.62	91%		2
Helping me decide to reduce outings to public places.	3.61	0.72	90%		3
Allowing me to monitor local and global news.	3.48	0.73	87%		4
Enabling me to share my knowledge about the virus with my family	3.45	0.77	86%		5
Enabling me to check up on the health and general welfare of family and friends.	3.42	0.74	86%		6
Providing entertainment programs such as TV shows and movies.	3.40	0.79	85%		7
Mitigating the effects of isolation from friends and colleagues.	3.37	0.77	84%		8
Enabling me to communicate with family and relatives.	3.32	0.85	83%		9
Informing me about the social conditions related to the virus and how to plan for the near future.	3.29	0.85	82%		10
Helping to avoid boredom during lockdown and quarantine	3.18	0.96	79%	Strong	11
Enabling me to share social life-related information with others.	3.16	0.94	79%		12
Providing help in identifying people in urgent need of help.	3.09	0.93	77%		13
Providing information about sources of food supply.	3.06	0.99	77%		14
Providing information about sources of medical supplies.	3.01	0.97	75%		15

The data presented above shows clearly that students felt that they gained significant social benefits from social media networks during the early period of the spread of COVID-19 (M = 50.15 and SD = 8.19). The mean relative strength score for social benefits was 0.84, which signifies a very strong endorsement. A wide range of social benefits was perceived; the sense of social responsibility they developed was ranked first and was exemplified in, for instance, the sharing of COVID-related knowledge with friends and family, the avoidance of behaviors which could spread the virus, along with a concern about checking up on family and friends and identifying those in the community who needed help. Social media also helped students to tolerate the effects of isolation and avoid boredom at a time when they were unable to enjoy their normal social interaction. There were also practical advantages such as knowing where to access food and medical supplies and treatment, both for themselves and for others, though these were ranked very slightly lower than other benefits.

### Survey section 6: educational benefits of social media use during the early stages of the pandemic

As explained earlier, soon after the WHO declared COVID-19 to be a pandemic, SQU, like many other educational institutions worldwide, moved from face-to-face to distance/remote/online learning, through electronic platforms and social media, with no idea of how long this situation would remain necessary. This transition was aimed at overcoming the effects of the emergency suspension, but the sudden change posed a number of challenges for both teachers and students. Still, both resorted to using the available social media networks and applications to facilitate teaching and learning and fulfil educational needs. (Study groups have been created on WhatsApp as well as on Facebook, and some recorded lessons have been provided via YouTube). Despite the need to adapt at very short notice, this remote learning was facilitated by the use of numerous technological tools including e-learning applications, web applications, television programs, online educational videos, live online lessons, virtual classrooms and of course programs on social media networks. These tools allowed teachers and trainers not only to provide educational content to students, but also to make it available anytime and anywhere; this helped students stay connected to the curriculum and complete their coursework on time despite the emergency situation. Table 7 below shows the various educational benefits of the use of social media during the early stage of the pandemic, according to students’ perceptions.

**Table 7:** Educational advantages of social media use during the early stage of the pandemic.

Educational benefits	Mean	Std. deviation	Relative mean	Benefits level	Ranking
Access to educational materials at any time.	3.68	1.06	92%	Very strong	1
Facilitating the uploading and submission of research and assignments for teachers.	3.55	1.45	89%		2
Following up on educational presentations and films provided by course instructors.	3.55	0.92	89%		3
Exchange of lectures and academic material with professors.	3.54	1.13	89%		4
Exchange of lectures and academic material with classmates	3.54	0.99	89%		5
Keep me in constant communication with all parties in the educational process.	3.50	1.11	88%		6
Provided communication channels for e-learning.	3.41	1.18	85%		7
Improved my knowledge about the study courses.	3.16	1.15	79%	Strong	8
Feeling reassured about my academic future.	2.94	1.55	73%		9

The results in this Table indicate that students perceived that social media were playing a valuable role in fulfilling their educational needs (M = 30.80 & SD = 7.89), with the average response at the top on the border between strong and very strong agreement. Students saw the most important educational benefits as the fact that social media use enabled them to access academic material at any time, as well as to submit research and academic assignments to their teachers. Social media, including WhatsApp and class WhatsApp groups, allowed constant and speedy communication between students and lecturers; students could also communicate with each other. Seven of the nine statements met with very strong agreement; that is, over 80% of respondents concurred. The last two statements received only slightly less agreement, and student confidence in their academic future, which could have been very much undermined by the move to remote learning, remained strong at 73%.

### Survey section 7: challenges of using social media during the early stage of the pandemic in Oman

While previous sections of the survey examined the positive effects of social media, this last investigates the difficulties involved in its use. The challenges noted by SQU student respondents in using social media during the early period of the pandemic can be broken into two clearly distinct areas: technical challenges and information-reliability challenges.

When the university and government imposed a suspension of “normal” education in an attempt to control the spread of the pandemic, this created an urgent shift from traditional education to distance and e-learning. As a result, there was an increased use of the social media platforms to which students and others had long grown accustomed to, and even dependent on. Indeed, the lockdown affected not only educational institutions but society in general and people sought more than ever to benefit from social media services to maintain communication and interaction. In Oman as in other societies, this increased the pressure on internet networks, which had little or no time to prepare to handle the much heavier demands on their services. At the same time, the consumers’ increased data usage meant higher costs, in a situation they had not willingly chosen.

Students expressed annoyance not only at the cost of using the internet but also complained about its slow speed, its inability to cover all areas of the country, the low efficiency of the networks, and the breakdowns both in the networks and in power supply, all of which affected the sustainability of communication. While it was true that.

Suddenly increased usage meant a deterioration in the quality and coverage of internet services in the country, people had complained even before the pandemic that service was often poor and could be intermittent in more remote areas.

The other area of challenge, the spreading of fake news, misinformation and exploitative messages, was nothing new; nor was the networks’ inability – or possibly unwillingness - to monitor and control it. The unprecedented nature of the pandemic, especially in its early stages when little was known even by the WHO and CDCs, meant that even reliable information and advice were frequently changing, and made deliberate or even unwitting sharing of inaccurate information particularly confusing and dangerous.

Students were strongly aware of all these challenges, and Table 8 below shows how they ranked their relative magnitude.

**Table 8:** Challenges of using social media during the stage of the COVID-19 pandemic.

Challenges of using social media	Mean	Std. deviation	Relative mean	Challenges level	Ranking
	3.42	0.79	86%	Very strong	1
Contradictory information published about the pandemic.	3.40	0.77	85%		2
High cost of subscribing to internet packages.	3.34	0.81	84%		3
Internet companies are not taking their social responsibility seriously.	3.30	0.83	82%		4
Unreliable sources of information published about the pandemic.	3.30	0.92	82%		5
Weak internet speed.	3.27	0.86	82%		6
Internet connection is erratic, with sudden breakdowns.	3.09	0.85	77%	Strong	7
Internet coverage not available in some areas.	2.89	0.94	72%		8
Deliberate marketing of false information about the pandemic.	2.88	0.84	72%		9
It is difficult to identify the location of those in need of help.	2.77	1.00	69%		10
Power outages leading to home internet disconnection.	2.37	1.14	59%	Average	11

Overall, students saw the challenges as significant, (M = 34.02, SD = 5.67), with a mean relative strength score of 77%, near the top of the survey’s accepted criteria for strong (strong ranging between 65% and 80%). Six of the eleven, however, were perceived as very strong; these were the most problematic and included both technical and informational issues. The highest-ranked statement highlighted the ease with which unverified and possibly inaccurate information could be shared on social media, a challenge repeated in the fifth-ranked statement. The cost of social media use was also high on the list, and the statement ranked fourth, “Internet companies are not taking their social responsibility seriously,” could refer both to pricing and to failure to monitor information-sharing, issues which will be explored further in later sections (Tables 9–12).

**Table 9:** *T*-test results in the responses of males and females regarding the dependent variables.

Dependent variable	Male mean	Female mean	*t*-value	*p*-value	Significance
The role of social networks in raising health-related issues during the early stage of COVID-19	39.26	42.15	−5.246	<0.001	Significant
Social benefits of social media networks during the early stage of COVID-19	48.79	51.16	−2.804	0.005	Significant
Educational benefits of social media network use during the early Stage of COVID-19	28.39	32.56	−5.202	<0.001	Significant
Challenges of using social media networks during the early stage of COVID-19	34.83	33.42	2.417	0.016	Significant

**Table 10:** *T*-test results measuring differences in responses to place of residence.

Dependent variable	Urban mean	Rural mean	*t*-value	*p*-value	Significance
The role of social networks in raising health-related issues during the early stage of COVID-19	41.73	39.98	3.134	0.002	Significant
Social Benefits of Social Media Networks during the early stage of COVID-19	50.75	49.75	1.534	0.126	Not significant
Educational Benefits of Social Media Network Use during the early Stage of COVID-19	29.73	32.08	−2.883	0.004	Significant
Challenges of using social media networks during the early stage of COVID-19	33.41	34.73	−2.262	0.024	Significant

**Table 11:** Results of one-way ANOVA analysis of income groups across the dimensions of social networking websites.

Dependent variable	Sum of square	*df*	Mean square	*F*	Sig.
The role of social networks in raising health-related issues during the early stage of COVID-19	874.56	3	291.52	10.500	<0.001
Social Benefits of Social Media Networks during the early stage of COVID-19	1763.75	3	587.92	9.347	<0.001
Educational Benefits of Social Media Network Use during the early stage of COVID-19	592.25	3	197.42	3.229	0.023
Challenges of using social media networks during the early stage of COVID-19	260.04	3	86.68	2.731	0.044

**Table 12:** Summary of statistically significant LSD *post hoc* comparisons by income level.

Dependent variable	Significant comparison(s)	Direction of difference	*p*-value
Health awareness role	Low vs. Avg/Above Avg/High	Higher in Avg & above	<0.001
Social benefits	Low vs. Avg/Above Avg/High	Higher in Avg & above	<0.001
Educational benefits	Low vs. Above Avg; Avg vs. Above Avg	Higher in Above Avg	*p* = 0.016, 0.011
Challenges	Low vs. Above Avg; Avg vs. Above Avg	More challenges in Low & Avg	*p* = 0.033, 0.026

*Differences were considered statistically significant at the 0.05 level.

### *T*-test results

The results of the t-test show statistically significant differences between males and females in all dependent variables. Females scored higher than males on the role of social media in raising awareness of health issues during the early stages of the COVID-19 pandemic, as well as on the social and educational benefits of using social media. Conversely, males scored higher than females on the challenges of using social media. These results suggest that females more highly valued the health, social, and educational roles of social media, while males expressed a higher level of awareness of the challenges associated with its use. All differences were statistically significant at the *p* < 0.05 level.

The results of the independent samples t-test showed statistically significant differences based on place of residence in three dimensions of the study. Students residing in urban areas had a higher average rating of the role of social media in raising awareness of health issues than those residing in rural areas (41.73 vs. 39.98, *p* = 0.002). Conversely, rural students scored higher averages on the educational benefits of using social media (32.08 vs. 29.73, *p* = 0.004) and on the challenges associated with its use (34.73 vs. 33.41, *p* = 0.024). No statistically significant differences were found between urban and rural students regarding social benefits (*p* = 0.126). The results indicate that place of residence was associated with different student perceptions of the health, educational, and challenges associated with social media, while their perceptions of its social benefits were similar.

The results of the one-way ANOVA showed statistically significant differences between the categories of the independent variable under comparison in all dimensions of the study. Differences appeared in the role of social media in raising health awareness (*F* (3,372) = 10.500, *p* < 0.001), in social benefits (*F* (3,372) = 9.347, *p* < 0.001), in educational benefits (*F* (3,366) = 3.229, *p* = 0.023), and in the challenges of use (*F* (3,372) = 2.731, *p* = 0.044). These results indicate that students’ perceptions differ among the categories studied. However, determining the categories between which the differences exist, and the direction of these differences requires post-hoc comparison tests.

Post-hoc comparisons using the LSD test revealed statistically significant differences based on income level across all dimensions of the study. In the health awareness dimension, the low-income group scored lower than all higher-income groups, and the middle-income group scored lower than both the upper-middle and upper-income groups. Regarding social benefits, the low-income group scored lower than all other groups. Educational benefits were higher among the upper-middle-income groups compared to the low- and middle-income groups. Concerning challenges, the low- and middle-income groups scored higher than the upper-middle-income group. These results indicate differences in the utilization of social media and the challenges associated with its use based on household income level.

### Open-ended questions

The survey ended with two open-ended questions; these asked for suggestions first about the potential use of social media to reduce the risks associated with COVID-19, and second for recommendations for community initiatives to improve the situation created by the pandemic. Participants put forward a wide range of practical and creative proposals. The most frequently mentioned issue related very closely to the key concerns in the Challenges section of the survey: first, the high cost of accessing the internet, along with its efficiency, and second, the need to ensure the reliability and accuracy of the pandemic-related information being disseminated.

Numerous participants recommended that internet costs be reduced, or that the internet and thus social media be made free and accessible to all. One student proposed, for example, that” Telecommunication companies must provide free unlimited internet until the crisis ends so that we can benefit from social media.” This message was voiced frequently, along with calls for internet speed and coverage to be increased. Equally common was the call for information about COVID-19 to be reliable, verified, controlled and ideally disseminated through one official or formal source to which everyone could refer and which everyone could trust. Alongside this were a number of calls for rumors to be responded to, for the community to be educated, and a few recommending laws to ban and/or “punish the sending of false messages related to the pandemic”.

There were also a number of recommendations about how to make COVID messaging more effective; these included providing the messages in different languages (presumably to reach the different nationalities working in Oman, and the different Omani and other language groups), creating messages appropriate for different age groups and segments of society, as well as making messaging sites more attractive by, for instance, making them interactive and using video and sound, establishing strong community groups and community information feeds, designing awareness publications based on interests such as health, sport etc., using celebrities, design experts, academics and the Fatwa Office to help spread COVID-related messages, and holding competitions with attractive prizes.

## Discussion

The present study demonstrates that social media played a multifaceted role during the initial stage of the COVID-19 pandemic in Oman, contributing to health awareness, social support, and educational continuity among university students while simultaneously presenting important technical and informational challenges. Rather than serving solely as communication platforms, social media became an essential component of students’ daily lives during the suspension of face-to-face teaching, enabling them to access health information, maintain social relationships, and continue their academic activities under unprecedented circumstances. This finding may be explained by the fact that social media represented one of the few communication channels capable of simultaneously supporting information exchange, educational interaction, and social connectedness during periods of mandatory physical distancing. These findings are consistent with previous studies showing that digital communication platforms became indispensable during the pandemic because they provided rapid access to information and facilitated interaction despite physical distancing measures (Abuhashesh et al., 2021; Elkhayat et al., 2022; Mohsen et al., 2022).

The findings can also be interpreted through the Uses and Gratifications Theory, which proposes that individuals actively choose communication media to satisfy specific information, educational, and social needs. During the early stage of the pandemic, students experienced considerable uncertainty regarding the disease and the sudden transition to remote learning, thereby making immediate access to reliable health and educational information particularly valuable. Consequently, social media became an attractive and readily accessible source for obtaining timely health information, communicating with peers and instructors, and supporting educational activities. The high levels of perceived health awareness, social support, and educational benefit reported in the present study therefore reflect the ability of social media to satisfy multiple needs simultaneously during a period of crisis rather than functioning merely as entertainment platforms.

The strong contribution of social media to health awareness is consistent with a growing body of international literature demonstrating that digital platforms became major channels for disseminating public health information during the COVID-19 pandemic. Students reported that social media increased their understanding of disease transmission, preventive measures, symptoms, and government regulations, suggesting that these platforms complemented official public health communication. Similar findings have been reported in studies conducted in Jordan and other countries, which concluded that social media significantly enhanced public awareness and encouraged adherence to preventive health behaviors during the pandemic (Abuhashesh et al., 2021; Mohsen et al., 2022). At the same time, the findings illustrate that students did not rely exclusively on social media without critical evaluation. Although social networking platforms represented the most frequently used source of COVID-19 information, participants expressed considerably greater trust in official government websites, government television, and the World Health Organization. This distinction between frequency of use and perceived credibility suggests that students were able to differentiate between accessibility and reliability, reflecting a relatively high level of digital and health information literacy during the crisis.

The findings concerning social support can be interpreted within the framework of Social Support Theory, which posits that social relationships and communication networks play a critical role in buffering the psychological impact of stressful events by providing emotional, informational, and interpersonal support. During the COVID-19 pandemic, restrictions on face-to-face interaction substantially increased students’ reliance on digital communication. In this context, social media enabled participants to maintain regular contact with family members, friends, and classmates while facilitating the exchange of health information, academic assistance, and emotional support. These findings suggest that social media functioned not merely as a communication technology but as an important mechanism for sustaining social connectedness and enhancing resilience during a period of considerable uncertainty.

This finding is consistent with a recent study that found that active use of social media was associated with increased perceived online response, which in turn was associated with increased perceived social support; and increased social support was associated with decreased loneliness and increased life satisfaction. This indirect pathway emerged during and after the lockdown (Yue et al., 2024).

Particularly noteworthy is the finding that students ranked the development of social responsibility toward family and society as the most important perceived social benefit. This finding is consistent with previous research indicating that, during public health emergencies, digital communication can strengthen community engagement and encourage young people to participate in collective responses that promote public well-being. Evidence from Rwanda demonstrated that youth volunteers were motivated to use community-based approaches to support governmental efforts in controlling COVID-19, highlighting the importance of social responsibility and civic participation during times of crisis (Bimenyimana et al., 2024).

This result extends beyond individual-level support and indicates that social media may also reinforce collective responsibility and community solidarity during public health emergencies. Such findings are likely to reflect important cultural characteristics of Omani society, where strong family relationships, social cohesion, and shared responsibility constitute fundamental social values.

This interpretation is further supported by recent evidence indicating that, during public health emergencies, social media can contribute to strengthening individual and community resilience by facilitating social connectedness, encouraging mutual support, and promoting collective responses to crises. Experiences reported among youth volunteers during the COVID-19 pandemic suggest that digital communication platforms extended beyond information sharing to foster adaptive coping and community engagement under conditions of prolonged uncertainty (Celestin et al., 2025).

Educationally, the findings indicate that social media made an important contribution to maintaining learning continuity following the sudden suspension of face-to-face teaching. Students particularly valued continuous access to educational materials, communication with instructors, collaborative knowledge sharing, and the submission of assignments through digital platforms. These findings are consistent with Khan et al. (2021), who demonstrated that social media became an effective environment for collaborative learning by supporting knowledge acquisition, knowledge sharing, peer interaction, and continuous communication between students and instructors during the COVID-19 pandemic (Khan et al., 2021).

This finding is also consistent with a recent study that showed that social media has enabled knowledge exchange and collaborative learning between students and teachers from different countries and highlighted its potential as a complementary tool in blended and hybrid learning environments during and after crises (Elenurm, 2025).

The positive evaluations reported by participants also suggest that the existing technological infrastructure at Sultan Qaboos University facilitated the rapid transition to remote learning despite the unexpected nature of the crisis. This finding may also reflect the university’s prior experience with digital learning platforms, which likely enabled both students and instructors to adapt more efficiently to emergency remote teaching. Nevertheless, the comparatively lower ranking of students’ confidence regarding their academic future indicates that maintaining educational continuity alone was insufficient to eliminate concerns about longer-term academic outcomes, highlighting the psychological uncertainty associated with the early stages of the pandemic.

An important finding of the present study is the apparent discrepancy between students’ patterns of social media use and their levels of trust in different information sources. Although social media platforms constituted the primary source of COVID-19-related information during the early stage of the pandemic, participants expressed substantially greater confidence in official government websites, government television, and the World Health Organization. This finding suggests that frequent use should not necessarily be interpreted as unquestioning acceptance of online information. Rather, students appeared to use social media primarily because of its accessibility and speed while simultaneously recognizing the greater credibility of official sources. This finding aligns with a recent study showing that individuals used social media selectively and cautiously during the pandemic, placing higher levels of trust in traditional and official sources. They also relied on the source’s reputation, credibility, and comparisons with other sources when evaluating information, underscoring the importance of media literacy in distinguishing between easily accessible and reliable information (Gies et al., 2024). The coexistence of high social media use and selective trust highlights the importance of strengthening digital health literacy so that users can critically evaluate information obtained from multiple online sources.

The study also identified several important challenges associated with social media use during the pandemic. Participants consistently emphasized the widespread dissemination of misinformation and contradictory information, together with concerns regarding internet costs, unstable network services, and privacy-related risks. These findings reinforce evidence from previous studies demonstrating that the COVID-19 pandemic was accompanied not only by a public health crisis but also by an “infodemic,” in which the rapid circulation of inaccurate or misleading information complicated public understanding and weakened confidence in health communication. A recent study from China supports the discussion of the challenges of misinformation; it showed that university students were aware of its negative effects, but most lacked the competence to recognize and verify it and expected a greater role from the government in limiting its spread (Jiang and Wang, 2024).

The prominence of misinformation in the present study further emphasizes the need for coordinated communication strategies involving governmental institutions, universities, and public health organizations to provide timely, reliable, and evidence-based information through the same digital platforms most frequently used by young adults.

Inferential analyses revealed differences in students’ perceptions based on gender, place of residence, and income level; females reported higher health, social, and educational benefits, while males reported greater challenges. Place of residence and income were also associated with varying levels of benefit and challenges, reflecting the impact of inequalities in access to digital resources.

The responses to the open-ended questions further support this interpretation. Rather than recommending restrictions on social media use itself, many students proposed improving the quality and credibility of online communication by establishing trusted official information channels, enhancing public awareness of misinformation, improving internet accessibility, and strengthening digital communication strategies. These recommendations indicate that participants viewed social media as a valuable communication resource whose effectiveness depends largely on the quality, credibility, and accessibility of the information being shared. This finding has practical implications for future public health emergencies, where official institutions may benefit from maintaining an active and visible presence on widely used social media platforms to counter misinformation and promote evidence-based health messages.

The timing of data collection should also be considered when interpreting the findings. The survey was conducted during the initial weeks following the transition to remote learning, when both students and academic staff were adapting to an unprecedented educational environment. The generally positive perceptions reported by participants may therefore partly reflect experiences during the early phase of this transition, when digital communication became essential for maintaining educational and social continuity. However, this interpretation should be viewed cautiously because the present study did not directly examine students’ attitudes toward the novelty of online learning or changes in their perceptions over time. Consequently, no causal conclusions can be drawn regarding whether these positive perceptions would have remained stable throughout the later stages of the pandemic. Indeed, subsequent studies conducted among university students in Oman have reported greater psychological stress and less uniformly positive experiences with prolonged online learning, suggesting that students’ perceptions may evolve as emergency conditions persist (Malik and Javed, 2021; Datt et al., 2022).

The present study has several strengths that enhance its contribution to literature. First, data were collected during the earliest stage of the COVID-19 pandemic in Oman, enabling the study to capture students’ experiences while major public health restrictions and the transition to remote learning were occurring. Second, the study examined health awareness, social support, educational continuity, and perceived challenges simultaneously, providing a comprehensive assessment of the multiple roles played by social media during a public health emergency. Finally, the study contributes evidence from the Omani context, where empirical research on students’ use of social media during the initial phase of the pandemic remains relatively limited.

Nevertheless, several limitations should be acknowledged. The cross-sectional design prevents conclusions regarding causal relationships or changes in students’ perceptions over time. The study also relied on self-reported responses, which may have been influenced by recall bias or social desirability bias. Furthermore, participants were recruited from a single public university, limiting the generalizability of the findings to students enrolled in other higher education institutions within Oman or elsewhere. Furthermore, although the questionnaire was distributed to all eligible undergraduate students, participation was voluntary and the final sample therefore represents a convenience sample. Consequently, the findings should be interpreted cautiously, as the participants may not fully represent the broader population of university students. In addition, the possibility of non-response bias cannot be excluded, as students who chose not to participate may have differed from respondents in their experiences or perceptions of social media use. Finally, the absence of longitudinal follow-up limited the ability to examine how students’ perceptions evolved throughout subsequent stages of the pandemic. Consequently, the findings reflect students’ perceptions during a unique historical period and may not fully represent patterns of social media use, trust in information sources, or educational experiences observed during later stages of the pandemic or in post-pandemic contexts. Future research employing longitudinal and multi-institutional designs would provide a more comprehensive understanding of the long-term educational, social, and health implications of social media use during public health emergencies.

### Implications and recommendations

The findings of the present study have important implications for higher education institutions, public health authorities, and professional disciplines that rely on digital communication during emergencies. The COVID-19 pandemic demonstrated that social media can play a valuable role in maintaining educational continuity, facilitating health awareness, and supporting students’ social connectedness during periods of major disruption. Universities should therefore consider integrating social media and digital communication strategies into their emergency preparedness and continuity plans. Beyond supporting online teaching, higher education institutions should strengthen students’ digital literacy skills, including the ability to critically evaluate online information, identify misinformation, and use social media responsibly for academic, health, and social purposes. Such initiatives would enhance institutional resilience and better prepare students for future public health emergencies and other crises.

The findings also have important implications for public health authorities and university decision-makers. Participants consistently emphasized both the benefits of social media for disseminating health information and the challenges associated with misinformation and unreliable online content. These findings highlight the importance of establishing coordinated digital communication strategies that provide accurate, timely, and evidence-based health information through the social media platforms most frequently used by young adults. Universities and public health organizations should work collaboratively to develop communication campaigns that are culturally appropriate, visually engaging, and tailored to the media consumption patterns of their target audiences. Such strategies may improve the effectiveness of health promotion efforts while reducing the spread of misinformation during future public health emergencies.

Although the participants represented students from multiple academic disciplines, the findings also offer relevant implications for social work education and professional practice. Social workers increasingly engage with individuals and communities through digital environments, particularly during disasters and public health crises. Consequently, social work education programs may benefit from incorporating training on the ethical and effective use of social media for health promotion, community engagement, digital communication, and crisis intervention. More broadly, interdisciplinary collaboration among social workers, health professionals, educators, and communication specialists is likely to strengthen the development of comprehensive digital support strategies capable of addressing the educational, psychosocial, and public health needs of diverse populations during emergency situations.

Finally, future research should extend the present findings by employing longitudinal and multi-institutional designs to examine how students’ perceptions of social media evolve throughout different stages of public health emergencies. Comparative studies across universities and cultural contexts would also provide a broader understanding of the factors influencing students’ trust in digital information sources, patterns of social media use, and the effectiveness of digital communication strategies in supporting learning, well-being, and health promotion.

## Conclusion

The current research is significant as it has examined the role of social media in enhancing health awareness, social support and educational progress among university students in Oman during the emergency created by the arrival of the Covid-19 pandemic in 2020. The study investigates both the positive impact of social media use, and also the challenges it poses, and thus provides a benchmark against which studies of the situation at later stages of the pandemic can be measured. It further highlights the potential of social work professionals to utilize these social media to achieve the preventive, therapeutic, and developmental objectives of their work, particularly during epidemics and disasters. It also suggests useful communication methods for public health officials and decision-makers, and the humanitarian community at large, to strengthen their communicative capacity through the use of social media, and thus to mitigate the impact of future disasters and crises.

## Data Availability

The original contributions presented in the study are included in the article/supplementary material, further inquiries can be directed to the corresponding authors.
